# Adipose Tissue-Derived Stem Cell Yield Depends on Isolation Protocol and Cell Counting Method

**DOI:** 10.3390/cells10051113

**Published:** 2021-05-05

**Authors:** Lukas Prantl, Andreas Eigenberger, Eva Brix, Sally Kempa, Magnus Baringer, Oliver Felthaus

**Affiliations:** Department of Plastic, Hand and Reconstructive Surgery, University Hospital Regensburg, Franz-Josef-Strauss-Allee 11, 93053 Regensburg, Germany; andreas.eigenberger@ukr.de (A.E.); eva.brix@ukr.de (E.B.); skempa@caritasstjosef.de (S.K.); mbaringer@caritasstjosef.de (M.B.); oliver.felthaus@ukr.de (O.F.)

**Keywords:** plastic surgery, adipose tissue, lipoaspirate, stem cells, fat grafting, cell yield, adipocyte viability

## Abstract

In plastic surgery, lipofilling is a frequent procedure. Unsatisfactory vascularization and impaired cell vitality can lead to unpredictable take rates in the fat graft. The proliferation and neovascularization inducing properties of adipose tissue-derived stem cells may contribute to solve this problem. Therefore, the enrichment of fat grafts with stem cells is studied intensively. However, it is difficult to compare these studies because many factors—often not precisely described—are influencing the results. Our study summarizes some factors which influence the cell yield like harvesting, isolation procedure and quantification. Stem cells were isolated after liposuction. Quantification was done using a cell chamber, colony counting, or flow cytometry with changes to one parameter, only, for each comparison. Quantification of cells isolated after liposuction at the same harvesting site from the same patient can vary greatly depending on the details of the isolation protocol and the method of quantification. Cell yield can be influenced strongly by many factors. Therefore, a comparison of different studies should be handled with care.

## 1. Introduction

Adipose tissue derived stem cells (ASCs) are mesenchymal stem cells (MSCs) with properties comparable to those of bone marrow stromal cells (BMSCs) [1]. They fulfill all criteria for MSCs proposed by the Mesenchymal and Tissue Stem Cell Committee of the International Society for Cellular Therapy [2]. ASCs grow plastic adherent in standard culture conditions and express surface markers CD44, CD73, and CD90 but not CD45 and HLA-DR. They have the potency to differentiate into osteoblasts, adipocytes and chondroblasts in vitro [3,4]. Additionally, it has been shown that ASCs can be induced to differentiate into myogenic, neurogenic, angiogenic, and hepatic lines [5,6,7,8,9,10]. However, the main advantage of ASCs over BMSCs is the possibility to harvest regenerative cells in much larger quantities with minimally invasive procedures [1,11].

The term fat grafting or lipofilling describes a procedure that is commonly applied in clinical practice to treat volume and contour defects in soft tissues [12]. Indeed, lipofilling is a common procedure in plastic surgery [13,14]. However, the graft stability following autologous fat transplantation remains unpredictable with absorption rates of up to 70% [15,16,17]. These high absorption rates are believed to be related to both reduced cell viability after liposuction associated stress and to lacking angiogenesis of the transplant [18,19]. Therefore, graft survival is closely related to cell viability after liposuction and subsequent vascularization of the grafted cells. It has been reported previously that the number and viability of grafted cells and their potential for neovascularization is important for the outcome of lipofilling [20,21,22,23,24]. Both problems could be solved by a higher number of ASCs because of their potential to replenish the cell number and because of their angiogenic properties [9,25,26,27]. Therefore, many studies aim to achieve an ASC-enriched lipoaspirate either by concentrating the aspirate or by supplementation with isolated ASCs (cell-assisted lipotransfer) [21,28,29].

Enzymatic digestion of fat tissue with collagenase lead to high ASC yield. Safety concerns about the manipulation of fat tissue cells and the use of xeno-origin enzymes necessitate the modification of digestion-free isolation methods for the purpose of routine patient treatment [30]. Whereas cutting, harvesting, or centrifuging represent only minimal manipulation of the cells, enzymatic digestion can be considered as a substantial manipulation with changes in cell characteristics and is therefore seen problematic by regulating authorities. Besides the cell manipulation, residual enzyme activity in the lipograft needs to be avoided. An abundance of studies has been published on various procedures to optimize ASC yield without enzymatic digestion, either by concentrating the lipoaspirate or by isolating the stem cells with mechanical procedures, only. However, in order to quantify the outcome of these efforts the use of collagenase still remains the standard method. Despite most authors apply comparable procedures of enzymatic digestion, results regarding stem cell yield vary greatly among these studies. This became evident when Locke et al. presented a review of studies that reported stem cell yields from adipose tissue ranging from 30,000 to 2,000,000 cells per mL lipoaspirate [11,31,32,33,34]. A review by Pak et al. published in 2017 reported a range from 500,000 to 2,000,000 nucleated cells per mL fat tissue with an ASC percentage ranging from 1% to 10% [35,36], resulting in a possible total range for ASCs from 5000 to 200,000 cells per mL. Obviously, this wide range has important implications for the comparison of reported stem cell yields of proposed novel enzyme-free stem cell enrichment procedures. Different counting method bear the problem, that different cell populations are compared with each other. Many, but not all plastic adherent cells from the stromal vascular fraction will be positive for MSC surface markers or will be colony-forming. Therefore, we utilize “cell yield” rather than “stem cell yield” when comparing different counting methods.

The objective of the study presented here is to compare the influence of variations of specific factors on the outcome of the cell yield to further improve lipotransfer procedures.

## 2. Materials and Methods

### 2.1. Adipose Tissue Harvest

Lipoaspirate from liposuctions was obtained from subcutaneous adipose tissue upon patients’ informed consent. Collection of lipoaspirate waste and isolation of cells had been approved by the Ethics Committee (08/117) of the University Hospital of Regensburg after patient’s informed consent. Liposuction was performed as described in the S2K guideline. Briefly, a 0.9% (*w*/*v*) solution of sodium chloride containing adrenaline (1:200,000) was infiltrated using a 2.5-mm injection cannula (Human Med AG, Schwerin, Germany) [37]. Liposuction was performed water jet–assisted with an even negative pressure of less than 0.5 mbar (Body-Jet, Human Med AG) using 3.8-mm cannulas (Human Med AG).

### 2.2. Lipoaspirate Handling

Lipoaspirate was either used directly after liposuction (control) or centrifuged for 5 min at 300 rcf. Blood and tumescence fluid were discarded and the lipoaspirate supernatant was further processed.

### 2.3. Stem Cell Isolation

ASC isolation was carried out as previously described [38]. Briefly, for enzymatic digestion the adipose tissue was mixed with an equal volume of alpha MEM (Sigma Alrich, St. Louis, MO, USA) and 10 µL per mL of a 100 U/mL collagenase (Collagenase from *Clostridium histolyticum* Sigma Blend Type L (Clostridiopeptidase A) in PBS solution (both Sigma Aldrich) and incubated for 45 min at 37 °C at constant shaking. Subsequently, the digested tissue is forced through a 100-µm filter (Merck-Millipore, Billerica, MA, USA) and centrifuged at 500 rcf for 5 min. The entire suspension except for the cell pellet containing the stromal vascular fraction (SVF) was discarded. In some cases, the cells were incubated with an erythrocyte lysis buffer (see below). Resuspended cells were either analyzed by flow cytometry (see below) or seeded into 6-well plates in cell culture medium (α-MEM (Sigma-Aldrich)) containing 20% heat-inactivated FBS (Pan-Biotech, Aidenbach, Germany), 2 mM glutamine (Thermo Fisher, Waltham, MA, USA), 100 U/mL penicillin, and 100 µg/mL streptomycin (both Sigma-Aldrich).

### 2.4. Erythrocyte Lysis

For erythrocyte lysis the cell pellet was resuspended in erythrocyte lysis buffer containing 155 mM NH_4_Cl, 10 mM KHCO_3_, and 0.1 mM EDTA in PBS (all Sigma Aldrich) and incubated for 6 min. Subsequently cells were centrifuged, and the supernatant was discarded. After a washing step with PBS, the cells were handled as the cells that had not been treated with lysis buffer.

### 2.5. Flow Cytometry

Flow cytometry was done as previously described [39]. Briefly, cells were washed with FACS-buffer (containing 0.01% sodium azide, 0.5% BSA, and 2 nM EDTA, all Sigma Aldrich) three times. For antibody incubation, cells were resuspended in 40 µL FACS-buffer supplemented with either 5 µL APC anti-human CD90 Antibody and 5 µL Alexa Fluor^®^ 488 anti-mouse/human CD44 antibody or their respective isotype controls (APC Mouse IgG1, κ Isotype Ctrl (FC) Antibody, Alexa Fluor^®^ 488 Rat IgG2b, κ Isotype Ctrl Antibody, all BioLegend, San Diego, CA, USA). After incubation on ice in the dark for 1 h, 1 mL FACS-buffer was added and the cells were centrifuged. The supernatant was discarded, and cells were resuspended in 500 µL FACS-buffer and measured using the FACS Canto II (BD Biosciences, Heildelberg, Germany). At least 50,000 events of each sample were recorded. Figures were created using FlowJo Version 10.7.1 (FlowJo LLC, Becton, Dickinson & Company, Franklin Lakes, NJ, USA).

### 2.6. Cell Counting

Cells seeded in 6-well plates were used for cell counting. Seeded cells were allowed to adhere for 24 h and afterwards washed thoroughly with PBS, disposing all non-adherent cells. Cell counting was done using two different methods. For the first method the cell equivalent of 1 mL lipoaspirate was seeded. After 24 h when the cells were given time to adhere but not to proliferate cells were washed thoroughly, fixed with 10% formalin, and stained with a 0.02% (*w*/*v*) crystal violet solution. Pictures from random sites of the plate surface were taken with the Wilovert S microscope (Helmut Hund GmbH, Wetzlar) and the ScopeTec DCM 800 camera, using the Scope Photo 3.0 software. The number of cells per cm^2^ was counted and projected to the complete well area. For the second method, a colony forming unit assay, the cell equivalent of 10 µL lipoaspirate was seeded to assure that single colonies can be observed. After adherence and washing, the cells were cultured for 14 days. Afterwards, cells were fixed with 10% formalin and stained with a 0.02% (*w/v*) crystal violet solution. The number of macroscopically visible colonies were counted and projected to the equivalent of 1 mL lipoaspirate which was used for adherence counting method. Results are shown as mean of five wells ± SD. To facilitate the direct comparison of parameter impact, results are shown as normalized values.

**Figure 1:**
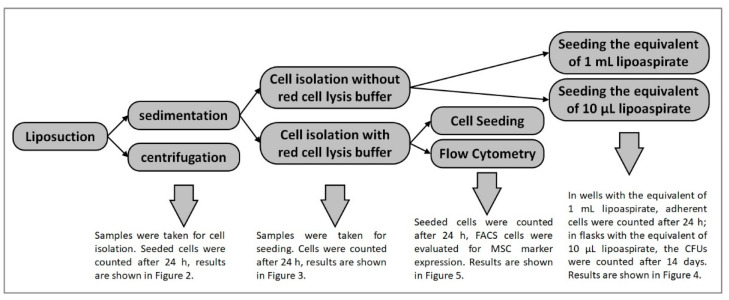
Schematic presentation of the experiment process. After liposuction the lipoaspirate is either allowed to sediment or centrifuged. An aliquot is taken from both samples and used for analysis of the parameter centrifugation. The rest of the sedimented lipoaspirate underwent the Adipose tissue-derived stem cell isolation protocol either with or without erythrocyte lysis buffer. An aliquot is taken from both samples and used for analysis of the parameter erythrocyte lysis. The rest of the cells isolated without lysis buffer are seeded in two different seeding concentrations and were analyzed for this parameter. The rest of the cells isolated with erythrocyte lysis buffer, which is a prerequisite for flow cytometry anyway, underwent either FACS analysis or were seeded as described above.

A schematic representation of the experimental process is shown in Figure 1.

### 2.7. Statistical Analysis

Student’s t-test was used for statistical analysis. *P*-values below 0.05 were considered statistically significant.

## 3. Results

### 3.1. Lipoaspirate Handling

**Figure 2:**
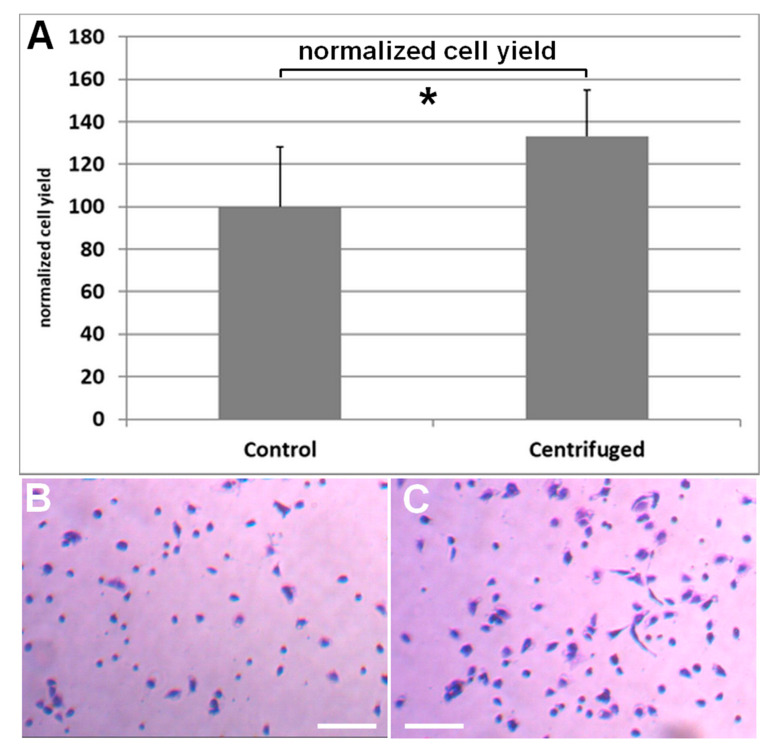
(**A**) Cell yield is increased 1.3-fold per mL when lipoaspirate is centrifuged directly after liposuction. For this comparison, the counting of adherent cells after 24 h was used. Results are shown as mean ± SD. Student’s t-test was used to assess statistical significance (*: *p* < 0.05). (**B**) Representative image of cells isolated from sedimented lipoaspirate. (**C**) Representative image of cells isolated from centrifuged lipoaspirate. The bar measures 100 µm.

Isolated cells from lipoaspirate that was either centrifuged directly after liposuction or unprocessed were seeded into 6-well plates and allowed to adhere. After 24 h the cells were washed thoroughly, fixed, stained, and counted under an inverse microscope. After lipoaspirate was separated from tumescence solution and free lipids by centrifugation, stem cell yield per milliliter lipoaspirate is increased 1.3-fold per mL (Figure 2).

### 3.2. Erythrocyte Lysis

**Figure 3:**
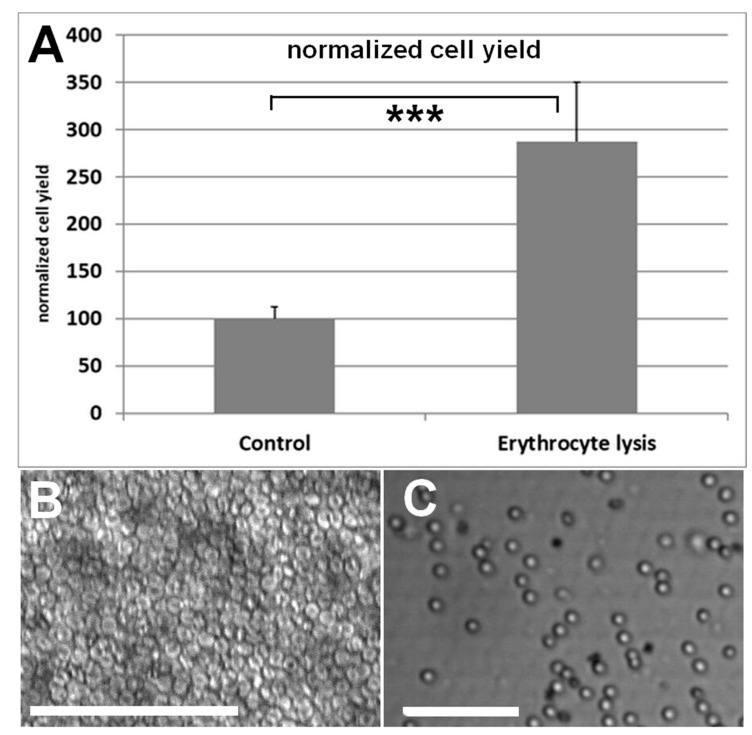
(**A**) Cell yield is increased more than 2.5-fold per mL when cells are incubated with an erythrocyte lysis buffer after stem cell isolation and prior to seeding. For this comparison, the counting of adherent cells after 24 h was used. Results are shown as mean ± SD. Student’s t-test was used to assess statistical significance (***: *p* < 0.001). (**B**) Representative image of seeded cells directly after seeding without red blood cell lysis buffer. The whole surface is covered with erythrocytes. (**C**) Representative image of seeded cells directly after seeding with red blood cell lysis buffer. Although many erythrocytes remain in the suspension, there is free surface area for the adipose tissue cells to adhere. The bars measure 100 µm.

Quantification was performed as described for the lipoaspirate centrifugation comparison. Directly after stem cell isolation and prior to seeding cells were incubated with an erythrocyte lysis buffer. Cell yield after treatment with isolation buffer was increased more than 2.5-fold (Figure 3).

### 3.3. Colony Forming Unit Assay

**Figure 4:**
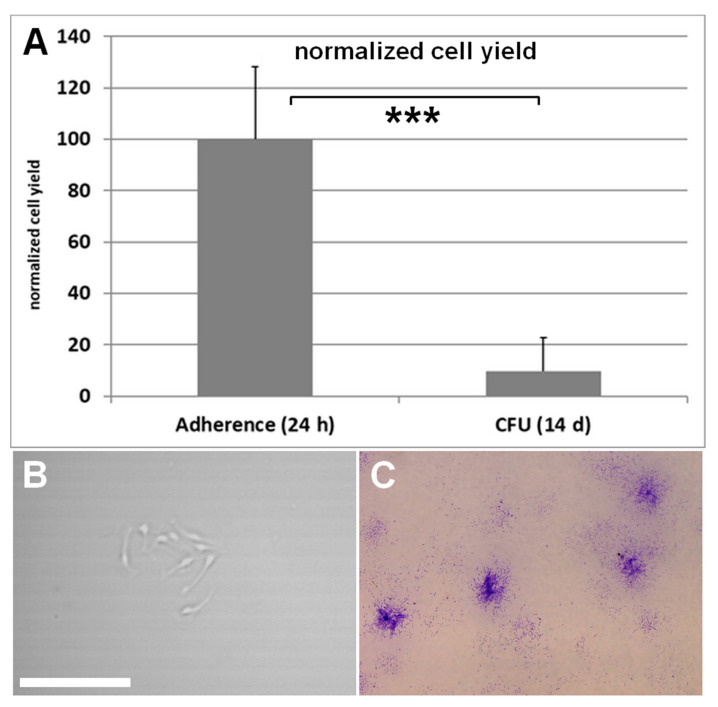
(**A**) Calculated cell yield is roughly 10 times higher when the adherent cell counting method is used compared to the colony forming unit assay. Results are shown as mean of five wells ± SD. Student’s test was used to assess statistical significance (***: *p* < 0.001). (**B**) A single colony after a few days of culture. The bar measures 100 µm. (**C**) The stained (crystal violet) colonies can be seen macroscopically after two weeks.

After the isolation the cell equivalent of either 1 mL or 10 µL lipoaspirate was seeded into 6-well plates. Adherent cells from the 1 mL equivalent were counted after 24 h, formed colonies from the 10 µL equivalent were counted after two weeks and multiplied by 100 (Figure 4).

### 3.4. Flow Cytometry Analysis

**Figure 5:**
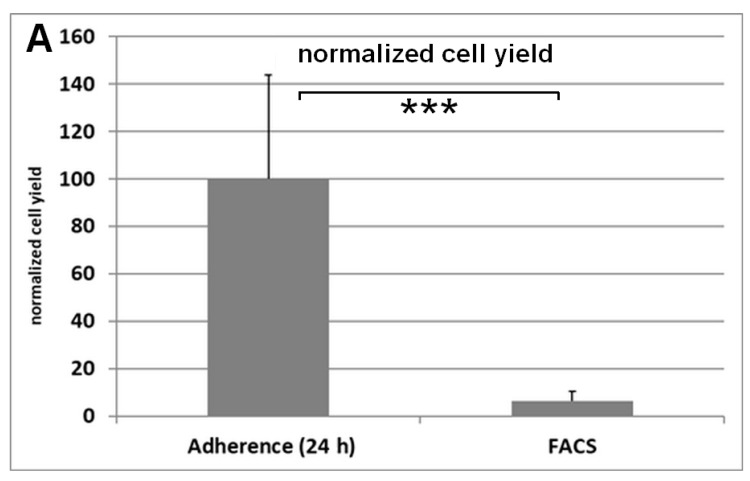
Calculated cell yield from the same lipoaspirate is decreased about 15-fold when detecting the CD44^+^CD90^+^-positive ASCs with flow cytometry instead of counting the adherent cells after 24 h. Results are shown as mean ± SD. Student’s test was used to assess statistical significance (***: *p* < 0.001).

**Figure 6:**
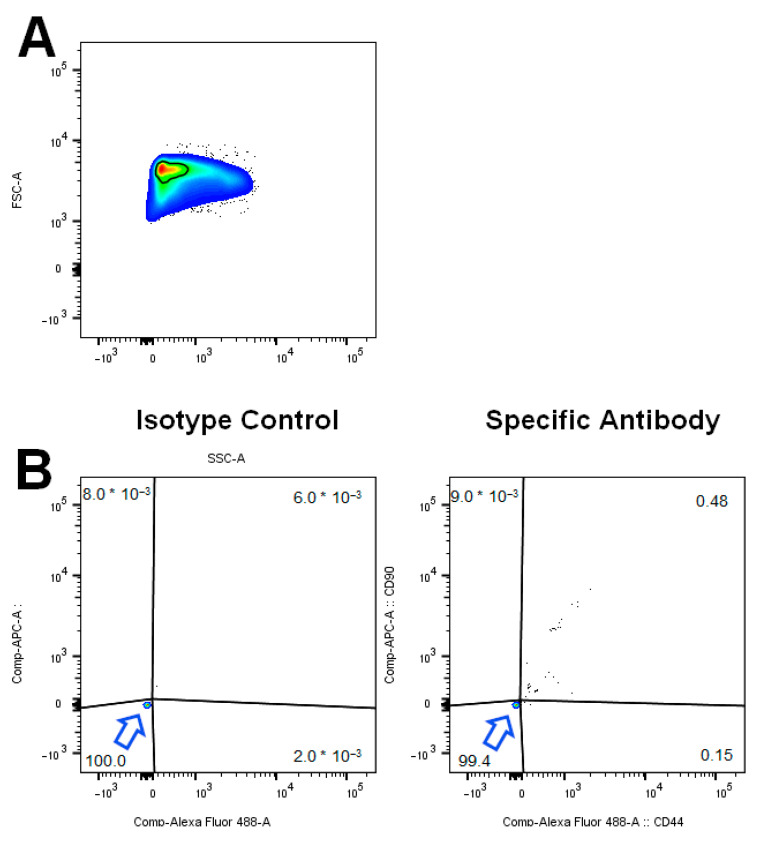
(**A**) Forward Scatter/Sideward Scatter Plot for cells isolated from adipose tissue. The gated cell population is shown. (**B**) Cells stained with isotype control antibodies are in the lower left quadrant of an APC/AlexaFluor488 Plot. For isotype control cytometry 50,000 events were counted (left). After staining with specific antibodies for CD44/CD90, nearly 0.5% of cells are positive for CD44/CD90. Arrows point to the main population. For specific antibody cytometry 100,000 events were counted. One of four measurements is shown. (right).

**Figure 7:**
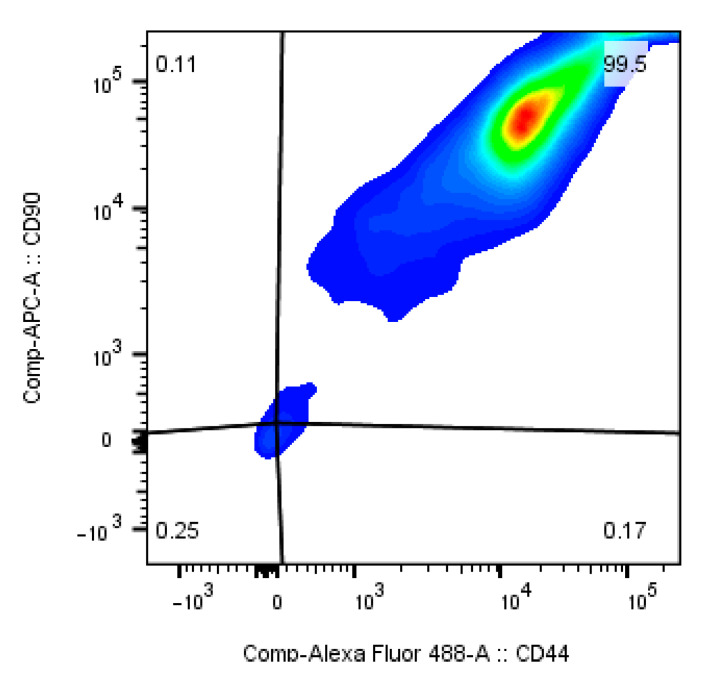
Cultured ASCs are positive for CD44 and CD90.

After the isolation process cells were either seeded into 6-well plates or underwent flow cytometry analysis. An aliquot of the cells for flow cytometry was used to calculate the total number of cells with a Neubauer chamber. Using the percentage of the CD44^+^CD90^+^-positive cells the number of ASCs was quantified. The quantification of the adherent cell counting method showed 15 times more cells than the ASCs detected by flow cytometry quantification (Figure 5). Only a small percentage of the cells resuspended from the SVF is positive for CD44 and CD90 (Figure 6). However, cells that are isolated from adipose tissue and cultured for several days on well plate surfaces with α-MEM are highly positive for CD44 or CD90 (Figure 7).

## 4. Discussion

High absorption rates are still considered the main concern in autologous fat transplantation [40]. Evidently, ASCs contribute to fat graft survival not only by their proliferative capacities but also by paracrine angiogenic properties [9,41]. Therefore, enrichment of lipoaspirate with ASCs is intensively investigated by numerous groups [36,42,43,44,45,46]. Safety concerns regarding enzymatic digestion of fat tissue have led to an increasing recognition of methods that circumvent the risks of enzymatic digestion [29]. Both the concern of substantial changes in cellular properties and residual collagenase activity have shifted the focus to enzyme-free stem cell enrichment processes for the clinical application. However, irrespective of the applied method enzymatic digestion remains the gold standard for the in vitro quantification of stem cells yields. An exceeding number of studies focus on stem cell isolation from adipose tissue. However, the results for stem cell yield vary greatly among studies, even without efforts to optimize stem cell content [34,36]. The objective of this study is to summarize parameters that influence the reported cell yield from fat tissue grafts and to evaluate the cell yield after varying single parameters during the isolation or quantification procedure.

It is believed that the number of ASCs can be influenced by donor demographics like age, gender, and BMI. Most studies report a negative correlation of BMI and ASC content per volume lipoaspirate [32,47], while other studies did not find significant differences [48]. Whereas most studies found no significant correlation between initial ASC yield and donor age [32,47,49] one study referring to a highly proliferative ASC subpopulation reported significant differences in ASC yield from older patients [50]. Other studies suggested that not ASC yield but ASC quality in terms of proliferation capacity and differentiation potential is impaired in older patients [51,52,53]. Similar observations exist for the correlation of ASCs and gender. The quantification method determines the difference in proliferation capacity and hence may as well influence graft survival. Apart from that, these findings have implications that should be considered when using fat tissue grafts for regenerative purposes. Essentially, neither demographic variables nor parameters may solely explain the broad range in stem cell yield found in the literature, but may contribute to this great variance, especially when cells are allowed to proliferate prior to quantification.

Whereas it is impossible for the surgeon to influence donor demographics, there are other outcome-related parameters during liposuction that may be controlled by the clinician: namely tissue-harvesting technique and tissue-harvesting site. Oedayrajsingh-Varma et al. did not find statistically significant differences in cell yield from abdomen, hip/thigh, and mamma tissue [54]. However, in this study only the number of nucleated cells was evaluated without further distinction. Fraser et al. reported a significantly higher yield of colony-forming cells from hip-harvested lipoaspirate than from abdomen-harvested fat tissue [55], whereas Jurgens et al. reported contrary results [56]. It is of note that the studies from both Oedayrajsingh-Varma et al. and Jurgens et al. compared different tissue harvesting sites from different patients whereas Fraser et al. harvested lipoaspirate from different sites of the same patient. Given the fact that donor demographics may influence the outcome, the comparison of harvesting sites within the same patient might be more reliable. The third outcome related parameter is the tissue-harvesting technique itself. When fat tissue resection, tumescent liposuction, and ultrasound assisted liposuction were assessed regarding post-harvesting cell viability, no statistically significant differences were found. Nevertheless, the resected tissue showed significantly higher yields of adherent proliferating cells [54]. Ozsoy et al. evaluated the influence of cannula sizes during the injection and aspiration process [57]. Injection cannula diameters of 1.5, 2.0, and 2.5 mm as well as aspiration cannula diameters of 2, 3, and 4 mm were investigated for this purpose. Whereas the size of injection cannula seemed to be of negligible impact, the lipoaspirate harvested with 4 mm aspiration cannulas yielded twice as many viable cells as the lipoaspirate harvested with the 2 mm aspiration cannula. Another study compared the conventional fat-harvesting technique as described by Coleman [58] to a novel micro-fat-harvesting technique. Although, no significant differences in initial cell yields were observed, cells harvested with the micro-fat technique showed significantly higher adherence. Again, depending on the quantification method, this can account for huge differences in the outcome.

The study presented here compared the influence of four distinct parameters on the cell yield: the lipoaspirate handling immediately after liposuction (centrifugation vs. sedimentation), specific details of the isolation protocol (erythrocyte lysis), as well as the quantification method (adherent cells vs. CFUs, microscopy vs. flow cytometry). Although the investigated cells were of the exact same adipose tissue origin, all of the 4 mentioned parameters variably contributed to differences in the observed cell yield. Firstly, centrifugation of the lipoaspirate lead to a 1.3-fold increase in adherent cell yield. Although this could be expected of more concentrated lipoaspirate, it illustrates that the comparison of total numbers from different studies is difficult. This phenomenon may as well confound results when lipoaspirate is allowed to sediment by gravity for different time spans. Regardless, precise information on centrifugation and duration of sedimentation is yet rarely considered in “Material and Methods” of published experiments.

The incubation with erythrocyte lysis buffer had a high impact on adherent cell yield. In fact, more than 2.5-fold more cells were detected with the adherent cell counting quantification when cells underwent erythrocyte lysis treatment prior to seeding. This might be explained by the fact that the vast majority of cells contained in lipoaspirate are erythrocytes and that these cells simply competitively interfere with attachment to the surface. Figure 3B,C illustrate that the free culture plate surface differs greatly with and without erythrocyte lysis. It is of note that studies that claim stem cell yields at the upper end of the broad range integrate a red blood cell lysis step into their isolation protocol [11,59]. In contrast, studies which report a stem cell yield at the lower end of that range tend to skip that step [31,32,60]. However, opposing examples can be found, and all these studies are distinguished by more than this parameter, rendering this comparison unreliable. Furthermore, it is important to notice that this might be just an artifact caused by the vast abundance of erythrocytes impairing the culture surface adherence of the adipose cells without any significance in a purely clinical setting. However, in this case it is also important to realize that differences in the observed laboratory cell yield caused by the utilization or the omission of erythrocyte lysis buffer would have no implication for lipografting.

The quantification method showed the biggest effect on the observed cell yield. Both the comparison of adherent cell counting method with colony forming unit assay method and the comparison of adherent cell counting method with flow cytometry analysis resulted in large difference in the observed cell yield.

The calculated cell yield is roughly 10 times lower when the number of colonies is the basis for the cell yield calculation. This is based on the fact that the capability of cells to adhere to the surface does not necessarily imply the capability to form colonies. Only a part of the cells of the SVF adhere to the cell culture surface, and of those, only a part can form colonies. This is in accordance with the reported frequency of stem cells in the stromal vascular fraction between 0.1% and 5% [57,61,62].

Measuring the stem cell yield by evaluating the percentage of CD44^+^CD90^+^-positive cells leads to a result that is 15 times lower than that reached with the adherent cell counting method. This cannot be explained with the fact that only a small percentage of the adherent cells express these surface markers. Flow cytometry with cells that were cultured for a few days after isolation are nearly 100% positive for these markers (Figure 7). One can only speculate about the reasons. The obvious explanation is that far more cells from the SVF are culture surface adherent than the CD44^+^CD90^+^ ASCs.

It is important to notice that the different cell yields observed after different quantification methods are in fact caused by the examination of different cell populations. However, this only emphasizes the conclusion that reported cell yields from different studies are difficult to compare.

## 5. Conclusions

There is consensus that there is a great clinical need to optimize stem cell yields with non-enzymatic methods during lipotransfer. However, donor demographics, harvesting site, harvesting method, lipoaspirate handling, isolation method, as well as the quantification procedure itself all have a strong impact on the observed cell enrichment. This should be taken into account when comparing different studies. Meta-analyses especially should be interpreted with care.

## Data Availability

Data sharing is not applicable to this article.
